# Effect on Health-related Quality of Life of changes in mental health in children and adolescents

**DOI:** 10.1186/1477-7525-7-103

**Published:** 2009-12-23

**Authors:** Luis Rajmil, Jorge A Palacio-Vieira, Michael Herdman, Sílvia López-Aguilà, Ester Villalonga-Olives, Josep M Valderas, Mireia Espallargues, Jordi Alonso

**Affiliations:** 1 Catalan Agency for Health Technology Assessment and Research (CAHTA), Roc Boronat 81-95 2nd Floor, Barcelona 08005, Spain; 2 Health Services Research Unit, Institut Municipal d'Investigació Mèdica (IMIM-hospital del mar), Dr Aiguader 88, Barcelona 08003, Spain; 3 CIBER en Epidemiología y Salud Pública (CIBERESP), Dr Aiguader 88, Barcelona 08003, Spain; 4 National Primary Care Research and Development Centre and NIHR School for Primary Care Research, University of Manchester, Williamson Building Oxford Road, Manchester M13 9PL, UK

## Abstract

**Background:**

The objective of the study was to assess the effect of changes in mental health status on health-related quality of life (HRQOL) in children and adolescents aged 8 - 18 years.

**Methods:**

A representative sample of Spanish children and adolescents aged 8-18 years completed the self-administered KIDSCREEN-52 questionnaire at baseline and after 3 years. Mental health status was measured using the Strengths and Difficulties Questionnaire (SDQ). Changes on SDQ scores over time were used to classify respondents in one of 3 categories (improved, stable, worsened). Data was also collected on gender, undesirable life events, and family socio-economic status. Changes in HRQOL were evaluated using effect sizes (ES). A multivariate analysis was performed to identify predictors of poor HRQOL at follow-up.

**Results:**

Response rate at follow-up was 54% (n = 454). HRQOL deteriorated in all groups on most KIDSCREEN dimensions. Respondents who worsened on the SDQ showed the greatest deterioration, particularly on Psychological well-being (ES = -0.81). Factors most strongly associated with a decrease in HRQOL scores were undesirable life events and worsening SDQ score.

**Conclusions:**

Changes in mental health status affect children and adolescents' HRQOL. Improvements in mental health status protect against poorer HRQOL while a worsening in mental health status is a risk factor for poorer HRQOL.

## Background

Mental health status has been shown to be significantly correlated with health-related quality of life (HRQOL) in both adult [1] and pediatric [2] populations. In fact, children with mental health problems have been reported to have poorer HRQL than children with physical disorders [3]. Likewise, children's mental disorders were shown to interfere significantly not only with their daily lives but with those of parents and families as well.

Most of the studies performed to date on the association between HRQOL and mental health in children have been cross-sectional [2-4]. Few, if any, studies have examined this association using a longitudinal design. In comparison with cross-sectional studies, longitudinal studies help to provide a clearer picture of the direction and magnitude of change in HRQOL, to identify factors associated with change over time, to identify particularly vulnerable populations or dimensions in which changes are most marked, and to confirm the results of cross-sectional studies. Determining the association between changes in mental health status and changes in HRQOL is important because it allows us to examine the extent to which improvements in psychopathology correspond to improvements in quality of life and, conversely, to investigate the effect of a deterioration or persistence of mental problems in terms of their impact on burden of disease.

The European KIDSCREEN study was conducted to develop a generic HRQOL questionnaire for children and adolescents 8-18 years old in 13 European countries [5]. In order to study the evolution over time of HRQOL measured using the KIDSCREEN instrument, a follow-up study was performed 3 years after the initial KIDSCREEN administration in the Spanish sample. One of the measures included at both study contacts was the Strengths and Difficulties Questionnaire (SDQ) which was designed to assess mental health status in the pediatric general population [6]. The inclusion of this questionnaire in the longitudinal study alongside the KIDSCREEN-52 instrument made it possible to study the degree of association between changes in mental health status and HRQOL.

The primary objective of the study was to assess whether changes in mental health status were associated with changes in HRQOL in children and adolescents aged 8 - 18 years. The potential mediating effect of gender and socio-economic status was also examined.

## Methods

### Sample and data collection

The Spanish KIDSCREEN baseline sample was recruited between May and November 2003 as part of the European KIDSCREEN fieldwork [5]. The target population for the KIDSCREEN study was children and adolescents aged 8-18. The aim was to recruit a sample that was representative by gender and 2 age groups (8-11 and 12-18 years old) in each participating country according to census data. Telephone sampling was performed centrally from Germany, and was carried out using a Computer Assisted Telephone Interview (CATI) with random-digital-dialing (RDD). Households were contacted by telephone and asked to participate by interviewers who had received study-specific training. If the family member contacted agreed to participate, the questionnaire and other study materials were mailed to the requisite address together with a stamped, addressed envelope for return of the completed questionnaire. A telephone hotline was used to provide further information about the survey. Two reminders were sent in cases of non-response (after two and five weeks) [7].

Between May and November 2006, follow-up questionnaires were posted by mail to all children/adolescents and their parents who had previously agreed to participate in the follow-up (n = 840 of 926 participants at baseline). The fieldwork followed the same methodology as used at baseline [7]. Postal reminders were sent four and eight weeks after the first mailing to those who had not returned their completed questionnaires. A third reminder was sent after twenty weeks and any remaining non-respondents were contacted by phone. Additionally, the proxy respondent who responded to the postal questionnaire was contacted at a later date by phone and asked to complete a psychiatric interview.

### Measures

HRQOL was measured at baseline and follow-up using the KIDSCREEN-52 questionnaire, a self-reported, generic measure of HRQoL for use in children and adolescents [8]. The KIDSCREEN-52 measures HRQOL in 10 dimensions: Physical Well-being (PH, 5 items); Psychological Well-being (PW, 6 items); Moods & Emotions (ME, 7 items); Self-Perception (SP, 5 items); Autonomy (AU, 5 items); Parent Relation & Home Life (PA, 6 items); Social Support & Peers (PE, 6 items); School Environment (SC, 6 items); Social Acceptance (bullying) (BU, 3 items), and; Financial Resources (FI, 3 items). The KIDSCREEN items use 5-point Likert-type scales to assess either the frequency (never-seldom-sometimes-often-always) or intensity (not at all-slightly-moderately-very-extremely). The recall period is 1 week.

Scores for each dimension are calculated using Rasch analysis [5] and then transformed into T-values with a mean of 50 and a standard deviation (SD) of 10. Higher scores indicate better HRQoL. KIDSCREEN-52 T scores refer to the mean values and SD from a representative sample of the European general population. In addition to the dimension scores, the KIDSCREEN-10 index was calculated as a global score for the longer version of the instrument [5]. The Spanish version of the KIDSCREEN-52 has been shown to have acceptable levels of reliability and validity [9].

As the KIDSCREEN was developed for use in population aged 8 - 18, both adolescent and parent versions of the scale were assessed for feasibility and relevance in population aged 19 - 21 years old in a pilot test (age range at follow-up of patients aged 16-18 at baseline, unpublished data). This confirmed that the instrument was applicable in this population.

Other variables collected in the present study were age, sex, family socio-economic status, and parental level of education. Socio-economic status was measured using the Family Affluence Scale (FAS) [10], which includes family car ownership, having their own unshared room, the number of computers at home, and how many times they spent on holidays in the past 12 months. FAS scores were categorized as low (0-3), intermediate (4-5), and high (6-7) affluence level. Socio-demographic information collected from parents included the highest family level of education according to the International Standard Classification of Education (ISCED) categorized as low (at most lower secondary level, ISCED 0-2); medium (upper secondary level, ISCED 3-4), and; high (university degree, ISCED 5-6) [11]. Baseline values for the FAS and Family level of education were used in the present analysis.

Children's mental health status was assessed using the Strengths and Difficulties Questionnaire (SDQ) collected from parents. The SDQ is a brief behavioural screening questionnaire for children and adolescents aged 4 - 16 that asks about their mental health symptoms and positive attitudes6. The instrument consists of 25 items measuring 5 dimensions of emotional symptoms, conduct problems, hyperactivity/inattention, peer relationship problems, and pro-social behaviour. All items are scored on a three point scale with 0 = not true, 1 = somewhat true, and 2 = certainly true. Higher scores indicate more problems except on the pro-social behaviour dimension. Items in the 4 problem dimensions are summed to give a total difficulties score ranging from 0 (no problems) - 40 (maximum problems). The SDQ has shown acceptable levels of validity in several studies [12-16]. The Spanish version has been shown to be reliable and valid [17]. Life events were assessed using the Coddington Life Events Scale (CLES) [18].

The instrument measures the occurrence during the previous year of 53 stressful life events. The impact of those events is measured in terms of Life Change Units (LCU) based on how recently and how frequently a particular life event occurred (more recent and repeated events are associated with higher scores). The Spanish version was recently adapted and shown to be reliable and valid [19].

### Statistical analysis

Sample characteristics at baseline and follow-up were calculated and compared using independent t tests or chi squared tests as appropriate. Respondents were classified into 3 categories according to differences between 2003 and 2006 on the SDQ total difficulties score. The 3 categories were those who scored below -1 Standard Deviation (SD) from the mean (improved), those who scored above +1 SD (worsened), and the remainder of the respondents (stable). Effect sizes (ES) between administrations for the 3 groups were calculated for KIDSCREEN-52 dimensions and the index, and ES of (0.2-0.5), (0.51-0.8) and (> 0.8) were considered small, medium, and large, respectively [20].

Ordinary least square regression models were used to estimate the effect of independent variables of interest (SDQ scores; life events) on KIDSCREEN-52 dimension scores and the index at follow-up. Only undesirable life events were included in the model as previous research had shown that these had a much stronger effect on HRQOL than other types of event [21]. We tested and discarded for co-linearity between independent variables before carrying out the multiple regression analysis. All models were adjusted by age, gender, socio-economic status, and KIDSCREEN score at baseline. Dependent variables were tested for normality before carrying out multivariate analysis. The level of statistical significance was set at 0.05 and analysis was adjusted for multiple comparisons using the Bonferroni method.

## Results

A total of 840 children and their parents participated at baseline and 454 at follow-up (response rate = 54%). Table 1 shows the sample characteristics at baseline and follow-up. When compared with non-respondents at follow-up, respondents were younger with a slightly higher parental level of education.

**Table 1:** Sample characteristics at baseline and participants at the Spanish KIDSCREEN follow-up study

	**N**	**(%)**	**Participants** **N (%)**	**Non-participants** **N (%)**	**p-value**	**Degree of freedom**
Gender						
Boys	420	50	218 (48%)	202 (52.5%)	0.213	1
Family Affluence Scale						
Low	167	20.2	83 (18.7%)	84 (22.1%)		
Middle	411	49.9	224 (50.5%)	187 (49.2%)	0.458	2
High	246	29.9	137 (30.9%)	109 (28.7%)		
Parental level of education						
Primary	356	43.2	177 (39.4%)	179 (47.7%)		
Secondary	195	23.7	119 (26.5%)	76 (20.3%)	0.032	2
University	273	33.1	153 (34.1%)	120 (32.0%)		
						
Age (mean, SD)	12.91 (2.91)		12.71 (2.88)	13.14 (2.92)	0.031	838
SDQ total difficulties (mean, SD)	8.14 (5.04)		7.89 (4.84)	8.43 (5.24)	0.130	809

**SDQ**: Strengths and Difficulties Questionnaire SD: Standard deviation

Missing values: FAS: 16; Parental level of education: 16; SDQ:16

Table 2 shows KIDSCREEN scores at baseline and follow-up and effect sizes between administrations for each of the 3 change categories studied (improved, stable, and worsened). In general, HRQOL deteriorated over time in all 3 categories and on almost all of the KIDSCREEN-52 dimensions and the index. Deterioration was much more marked in the groups classified as 'worsened' in almost all KIDSCREEN dimensions and index, ranging from an ES = -0.81 (PW) to 0.18 (BU). The group classified as 'improved' showed the smallest overall reduction in HRQOL, ranging from an ES = -0.26 (AU) to 0.51 (BU). The 'stable' group started above the 50 score which reflects the European average, and showed minimal to moderate deterioration at the end, ranging from ES = -0.35 on Self-perception to 0.35 in Bullying. The "improved' group started below 50 mainly in physical and psychological dimensions and showed even slightly higher scores than the 'stable' group at follow-up. The only KIDSCREEN-52 dimension to show improvement was the bullying dimension, with improvement being observed in all three groups.

**Table 2:** Mean Scores on the KIDSCREEN-52 dimensions and Index according to the 3 analyzed categories in the Strengths and Difficulties Questionnaire (SDQ) at baseline and in the follow-up, and effect size (ES)

		**PH**	**PW**	**ME**	**SP**	**AU**	**PA**	**PE**	**SC**	**BU**	**FI**	**INDEX**
**Improvement (n = 43)**												
Follow-up		48.9	50.7	52.2	46.8	51.3	51.8	51.8	52.6	53.7	52.5	52.6
Baseline		49.2	49.3	49.7	48.6	54.2	52.3	53.6	51.5	48.3	50.9	52.4
Effect size		-0.02*	0.14*	0.21*	-0.15	-0.26*	-0.04	-0.17	0.09	0.51*	0.16	0.01
**Stable (n = 339)**												
Follow-up		48.9	50.0	50.8	47.0	50.3	50.1	51.7	50.8	52.5	52.4	50.3
Baseline		52.4	53.0	51.9	50.0	52.0	52.2	54.6	52.7	49.1	51.3	53.7
Effect size		-0.33*	-0.32*	-0.10	-0.35*	-0.19	-0.23*	-0.32*	-0.18	0.35*	0.13	-0.34*
**Worsened (n = 48)**												
Follow-up		44.9	45.2	44.4	46.0	46.4	46.6	48.5	47.0	51.7	48.8	45.3
Baseline		50.6	53.1	48.9	50.4	52.2	48.8	53.8	52.5	49.9	50.6	51.0
Effect size		-0.50*	-0.81*	-0.44*	-0.46*	-0.53*	-0.19	-0.52*	-0.58*	0.18	-0.17	-0.56*

**PH: **Physical Well-being, **PW: **Psychological Well-being, **ME: **Moods & Emotions, **SP: **Self-Perception, **AU: **Autonomy, **PA: **Parent Relation & Home Life, **PE: **Social Support &Peers, **SC: **School Environment, **BU: **Social Acceptance **FI: **Financial resources

**SDQ **was categorized according to differences in the total difficulties scores from parents 2006 -- 2003: improvement (below -1 Standard Deviation, SD; stable (-1 to +1 SD); or worsened (above +1 SD).

*ES statistically significant at p < 0.05.

Table 3 shows the results of the linear regression model used to analyze the association between mental health variables and HRQOL scores in 2006 after adjusting by socio-demographic variables and baseline HRQOL. The two factors with the strongest influence on HRQOL scores in 2006 were the SDQ 'worsened' category and undesirable life events in the past 3 years. For example, an undesirable life event such as breaking up with a boyfriend/girlfriend would be associated with a decrease of (weighted life event units = 39* [-1.7]/10) 6.6 points on the KIDSCREEN School Environment dimension (between 0.5 and 1 standard deviation). SDQ category of 'worsened' most strongly influenced the dimensions of psychological well-being, moods and emotions, autonomy and parents. Notably, undesirable life events were associated with deterioration on all HRQOL dimensions except that of Peers, which showed a statistically significant improvement. R^2 ^ranged from 0.16 to 0.33. No significant differences were found after stratifying the sample by age and gender.

**Table 3:** Multiple regression analysis of KIDSCREEN-52 dimensions and the KIDSCREEN-10 Index at follow-up

		**PH**	**PW**	**ME**	**SP**	**AU**	**PA**	**PE**	**SC**	**BU**	**FI**	**Index**
**(Constant)**		30.2*	31.3*	29.5*	32.5*	39.9*	30.7*	39.0*	33.6*	38.0*	40.1*	36.2*
**Age**		-0.4	-0.1	0.1	-0.2	-0.5*	0.2	-0.2	0.0	-0.1	-0.4	-0.2
**Gender**		2.4	-0.1	0.9	3.0*	0.5	-0.5	-3.1*	-1.1	-0.8	-0.9	0.3
**FAS**												
**Low**		0.3	0.0	1.0	1.6	2.0	-1.0	1.7	-0.9	-0.5	-3.0	0.8
**Medium**		0.4	-0.2	0.3	0.8	0.0	-1.0	1.0	0.0	-0.1	-1.8	0.6
**Parental education**												
**Primary school**		-0.6	1.0	1.0	0.7	0.4	1.0	-2.1	0.2	0.2	-0.7	-0.6
**Secondary school**		-1.0	0.9	0.8	0.3	-0.1	0.1	-2.6	-0.8	0.3	-0.8	-0.3
**Kidscreen Baseline score**		0.4*	0.4*	0.4*	0.2*	0.3*	0.4*	0.4*	0.4*	0.3*	0.4*	0.3*
**SDQ**												
**Improvement**		1.4	1.9	2.2	-0.5	0.0	1.5	0.6	2.3	1.3	0.3	3.0
**Worsened**		-3.2	-4.6*	-5.2*	-1.1	-3.9*	-1.9	-3.0	-2.9	-0.6	-2.4	-4.0*
**Undesirable life events**		-1.2*	-1.2*	-1.0*	-0.3	-0.1	-1.4*	0.9*	-1.7*	-0.7	-0.6	-1.3*
											
**R**^**2**^		0.31	0.25	0.21	0.20	0.14	0.19	0.20	0.26	0.15	0.25	0.31

**PH: **Physical Well-being, **PW: **Psychological Well-being, **ME: **Moods & Emotions, **SP: **Self-Perception, **AU: **Autonomy, **PA: **Parent Relation & Home Life, **PE: **Social Support &Peers, **SC: **School Environment, **BU: **Social Acceptance **FI: **Financial resources.

**FAS**: Family Affluence Scale

**SDQ**: Strengths and Difficulties Questionnaire;

**Reference category**: Gender: female; FAS: low family affluence; Parental level of education: university degree; SDQ: stable.

*Statistically significant beta coefficients after correction for multiple comparisons (p < 0.005).

## Discussion

We found that HRQOL worsened as a whole in this sample of children and adolescents followed over 3 years, but that the decline was much more marked in those whose mental health deteriorated. In those whose mental health remained stable or improved over the study period, the decline in HRQOL was relatively slight. Regression modelling showed that current HRQOL was more influenced by worsening mental health and undesirable life events in the past 3 years than by the other factors included.

The study had some limitations. Firstly, the response rate at follow-up was only 54% and there were some differences between participants and non-participants. As a consequence, a selective follow-up could have biased our assessment of the evolution of HRQOL. Nevertheless, the response rate was similar to that in other longitudinal population-based studies [22]. Moreover, although those followed up were slightly younger and from more educated families than non-participants, there were no differences in their baseline KIDSCREEN-52 scores. Secondly, the 3 year interval between baseline and follow-up and the fact that there was only one follow-up administration makes it difficult to establish trends, as many intervening events may not be captured. Nevertheless, this is one of few studies to attempt to determine the impact of changes in mental health on HRQOL in a longitudinal design and it provides some indication of directionality. Future studies should consider more, and more frequent study contacts. One further major limitation of this study is that it was not possible to perform structured clinical interviews, and results and conclusion were based on self and parents' reports data by mails. Probably this will significantly decrease the validity of the data given the absence of psychiatric diagnosis. Nevertheless, the SDQ has been widely used as a screening tool and has been shown to be reliable and valid in detecting possible cases of psychopathology [6,16]. It has also been proposed as one of the principal tools to be used worldwide for screening purposes as well as in clinical assessment and cross-cultural comparisons [23]. Finally, the SDQ and the KIDSCREEN-52 were originally intended for use in age groups 4 - 16 and 8 - 18, respectively, and here they were used in subjects who were above the upper age limit at follow-up. This may have affected results, though cognitive debriefing in older respondents suggested that the instruments were acceptable and relevant in participants aged 16 - 21 (unpublished data).

Strengths of the present study include the fact that it is one of the first to examine correlations between mental health and HRQOL in a longitudinal design, thus adding to knowledge contributed by earlier cross-sectional studies. For example, we have shown that HRQOL in this age group declines in general, but that the decline is exacerbated in those whose mental health also worsened. Improvements in mental health appear to act as a protective factor against this overall decline. The overall deterioration observed is likely due to pubertal changes which were shown to lead to deterioration in HRQOL in a previous study [24]. A further strength of the study was the use of well-validated instruments to measure HRQOL and mental health. The Spanish versions of the KIDSCREEN-52 [9] and the SDQ [18] showed good reliability and validity. For HRQOL measures, it is considered preferable to use self-report whenever feasible [25]. Likewise, we obtained ratings of mental health from parents and used a 1-SD cut point on the measure, thereby applying quite a strict definition of change on mental health, but one which ensures a high level of sensitivity. It is, for example, higher than the 0.8 SD from the mean, which is generally accepted as representing a large change in patient-reported outcome measures [26,27]. Moreover, it likely represents an improvement over previous strategies used to classify children as cases or non-cases and which showed relatively low ability to detect cases [16].

The findings are in line with those of other studies which have shown that children with mental disorders have significantly worse HRQOL than children with no such disorders and that they often have worse HRQOL than children with physical disorders [3]. Other studies in this area have also shown that specific HRQOL sub-domains are associated with distinct diagnostic categories such as attention-deficit and disruptive disorders, anxiety disorders, pervasive developmental disorder and others [4]. Longitudinally designed studies to assess the impact of mental health problems on HRQOL have primarily focused on analyzing changes in specific symptoms or clinical outcomes in diseases such as attention deficit hyperactivity disorder in samples of children attending health services [28,29]. A cross-sectional study of the relationship between mental health and HRQOL in representative samples of children and adolescents from 12 European countries found a moderate effect size (range 0.21 in Switzerland to 0.44 in the Czech Republic) on the KIDSCREEN-10 index when children categorized as without mental health problems were compared with probable cases [30]. These results are similar to those from the BELLA study, a longitudinal study to collect information on mental health and HRQOL in a representative sample of German children and adolescents and their parents [31]. Although only cross-sectional baseline data are currently available, the authors also found lower HRQOL in children classified as a probable or possible case of mental health problems on the SDQ compared with healthy children. Again, the HRQOL of these children was found to be poorer than that of children with special health care needs, or who reported pain or asthma [32]. In the present study we did not collect data on other specific physical symptoms or diagnoses. Nevertheless, our study confirms that persistence of poor mental health or worsening in mental health status may have a multidimensional effect on HRQOL 3 years later.

The results of the present study also adds to earlier findings that one of the better predictors of current psychopathology was previous psychopathology [33,34] in the sense that those who had psychopathology at any point in the study had poorer HRQOL than those without psychopathology at any point in the study. This indicates the considerable importance of mental health on HRQOL even when mental health improved over the study period. The apparently paradoxical association between the occurrence of undesirable life events and an improvement in HRQOL in the Peers and Social Support dimension could be explained by the type of life event. For example, the break-up of a romantic relationship could have a positive impact on other peer relationships.

Future research should address specific mental health problems such as attention deficit and hyperactivity and emotional problems to determine the extent to which changes in symptoms over time have a corresponding effect on HRQOL and whether HRQOL after treatment interventions recovers to the level of the general population. Future studies of specific, medium or long-term interventions (behavioural or other types of therapy) should also take into account that the natural course of HRQOL in this population is to decline so benefits from any interventions in terms of HRQOL might not be as large as initially expected.

## Conclusions

Changes in mental health status can affect HRQOL over time in children and adolescents. An improvement in mental health status appears to protect against the general deterioration seen in this group as a whole, whilst worsening mental health is a risk factor for more severe deterioration in HRQOL.

## Competing interests

The authors declare that they have no competing interests.

## Authors' contributions

LR, JMV, ME and JA participated in the conception and design of the study. LR, JAPV, and EVO analyzed the data. MH and LR participated in the drafting of the article. All authors contributed to a critical revision of the manuscript and made a substantial contribution to its content, and read and approved the final manuscript.
